# Protopine/Gemcitabine Combination Induces Cytotoxic or Cytoprotective Effects in Cell Type-Specific and Dose-Dependent Manner on Human Cancer and Normal Cells

**DOI:** 10.3390/ph14020090

**Published:** 2021-01-26

**Authors:** Mercedes Garcia-Gil, Benedetta Turri, Morena Gabriele, Laura Pucci, Alessandro Agnarelli, Michele Lai, Giulia Freer, Mauro Pistello, Robert Vignali, Renata Batistoni, Silvia Marracci

**Affiliations:** 1 Department of Biology, University of Pisa, 56127 Pisa, Italy; mercedes.garcia@unipi.it (M.G.-G.); bene.turri@gmail.com (B.T.); A.Agnarelli@sussex.ac.uk (A.A.); robert.vignali@unipi.it (R.V.); renata.batistoni@unipi.it (R.B.); 2 Interdepartmental Research Center “Nutraceuticals and Food for Health”, University of Pisa, 56127 Pisa, Italy; 3 Institute of Agricultural Biology and Biotechnology, National Research Council, 56124 Pisa, Italy; gabriele@ibba.cnr.it (M.G.); pucci@ibba.cnr.it (L.P.); 4 Retrovirus Centre, Department of Translational Medicine and New Technologies in Medicine and Surgery, University of Pisa, 56127 Pisa, Italy; michele.lai@unipi.it (M.L.); giulia.freer@unipi.it (G.F.); mauro.pistello@unipi.it (M.P.); 5 Istituto Nazionale per la Scienza e Tecnologia dei Materiali, 50121 Florence, Italy

**Keywords:** protopine, gemcitabine, cytotoxicity, cytoprotection, cell cycle, ROS

## Abstract

The natural alkaloid protopine (PRO) exhibits pharmacological properties including anticancer activity. We investigated the effects of PRO, alone and in combination with the chemotherapeutic gemcitabine (GEM), on human tumor cell lines and non-tumor human dermal fibroblasts (HDFs). We found that treatments with different PRO/GEM combinations were cytotoxic or cytoprotective, depending on concentration and cell type. PRO/GEM decreased viability in pancreatic cancer MIA PaCa-2 and PANC-1 cells, while it rescued the GEM-induced viability decline in HDFs and in tumor MCF-7 cells. Moreover, PRO/GEM decreased G1, S and G2/M phases, concomitantly with an increase of subG1 phase in MIA PaCa-2 and PANC-1 cells. Differently, PRO/GEM restored the normal progression of the cell cycle, altered by GEM, and decreased cell death in HDFs. PRO alone increased mitochondrial reactive oxygen species (ROS) in MIA PaCa-2, PANC-1 cells and HDFs, while PRO/GEM increased both intracellular and mitochondrial ROS in the three cell lines. These results indicate that specific combinations of PRO/GEM may be used to induce cytotoxic effects in pancreatic tumor MIA PaCa-2 and PANC-1 cells, but have cytoprotective or no effects in HDFs.

## 1. Introduction

Combination of natural compounds with conventional cancer chemotherapy has shown promising outcomes, due to their capability of enhancing the anticancer efficacy without increasing toxicity on normal tissues [1,2,3]. The chemotherapeutic drug gemcitabine (GEM) (2′,2′-difluorodeoxycytidine) is a nucleoside analogue of cytidine, approved for the treatment of various carcinomas, including pancreatic ductal adenocarcinoma (PDAC) and breast cancer, either as a single agent or in combination with other chemotherapeutic drugs [4,5,6]. GEM is incorporated into DNA during replication and inhibits DNA chain elongation; in this way, it exerts cytotoxic effects on dividing cells, producing multiple side-effects including myelosuppression, thrombocytopenia, edema and cutaneous toxicity [7,8]. GEM is also able to increase oxidative cellular stress and this is considered one of the mechanisms for its antitumor activity [9]. Unfortunately, development of chemoresistance often occurs, reducing GEM’s efficacy [10,11]. Identification of compounds that can enhance GEM antitumor effects, by minimizing its cytotoxicity on normal cells and/or by helping to overcome the chemoresistance to the drug, has started to be addressed for the successful treatment of tumors [2,11,12]. Protopine (PRO) is a benzylisoquinoline alkaloid present in several plants, including Papaveraceae and Fumariaceae [13], and is widely used in traditional medicine [14,15,16]. Like other natural alkaloids [17,18], PRO exhibits many pharmacological properties, such as anti-inflammatory [19], hepatoprotective [20], antiparasitic [21] and antimicrobial activities [22]. More recently, PRO was shown to exert anticancer effects [23,24,25], promoting abnormal assembly of the mitotic spindle and apoptotic cell death in human prostate cancer cells [23], antiangiogenic effects in human umbilical vein endothelial cells (HUVECs) and antiadhesive/invasion properties in MDA-MB-231 breast cancer cells [23,24]. Conversely, PRO showed cytoprotective effects in rat pheochromocytoma PC12 cells, increasing cell viability and enhancing antioxidant mechanisms [26]. In this paper, we have studied the effects of PRO, alone or in combination with GEM, on different human tumor cells, using human dermal fibroblasts (HDF_S)_ as non-tumor cells. We show that PRO, alone or in combination with GEM, exerts cytotoxic or cytoprotective effects in a cell type-specific and dose-dependent manner.

## 2. Results

### 2.1. Effect of PRO on Cell Viability of U343, U87, MIA PaCa-2, PANC-1, MCF-7 Cells and HDFs

**Figure 1:**
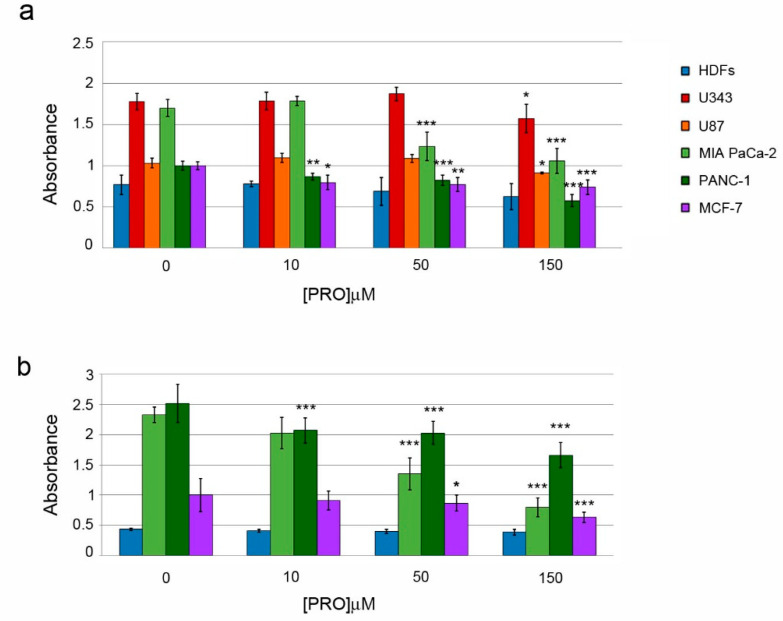
Effects of protopine (PRO) on cell viability. (**a**) Cell viability of human dermal fibroblasts (HDFs), U343, U87, MIA PaCa-2, PANC-1 and MCF-7 cells was evaluated after treatments for 48 h with different PRO doses, by using MTT. Graph columns represent mean of absorbance values ± standard deviation (SD) measured with MTT assay. In (**b**) Graph columns represent mean of absorbance values ± SD evaluated with crystal violet (CV) assay in the indicated cell lines. Graph columns represent mean ± SD of fluorescence intensity values. Control group contained only PRO vehicle, DMSO. The PRO doses (μM) used in treatments are indicated in x-axis. The symbols * indicate statistical significance versus control group. * *p* < 0.05; ** *p* < 0.01; *** *p* < 0.001.

We first examined the impact of PRO (3 nM–300 μM) on viability of U343, U87, MIA PaCa-2, PANC-1, MCF-7 and HDF_S_ by using the viability assays described in Materials and Methods. Compared to control cells treated only with the vehicle dimethyl sulfoxide (DMSO), PRO decreased viability in HDFs only at high concentrations (200–300 μM) (Figure S1). PRO may exert a hormetic effect on viability of PANC-1 cells: a low dose of PRO (0.016 μM) significantly increased viability, while higher doses (10–150 μM) decreased it (Figure S1).We found that in MTT (3-(4,5-dimethylthiazol-2-yl)-2,5-diphenyltetrazolium bromide) assay, 10 μM PRO significantly decreased viability in PANC-1 and MCF-7 cells (to 74.0 ± 5.2% and 72 ± 7%, respectively); an increase to 50 μM PRO reduced viability also in MIA PaCa-2 cells (to 73 ± 10%); a further increase to 150 μM PRO was also able to decrease viability in glioblastoma U343 and U87 tumor cells (to 92 ± 10% and 88 ± 1%, respectively), but not in HDFs (Figure 1a). Similar results were obtained with the crystal violet (CV) staining method (Figure 1b). These results overall indicate that PRO exerted higher cytotoxicity in pancreatic adenocarcinoma MIA PaCa-2 and PANC-1 cells, as well as in breast cancer MCF-7 cells, than in glioblastoma tumor cell lines U343 and U87 (Figure 1a). Notably, HDFs were more resistant to PRO than the analyzed tumor cell lines (Figure 1a,b).

### 2.2. Impact of PRO/GEM Combination on the Cell Viability of MIA PaCa-2, PANC-1, MCF-7 and HDFs

**Figure 2:**
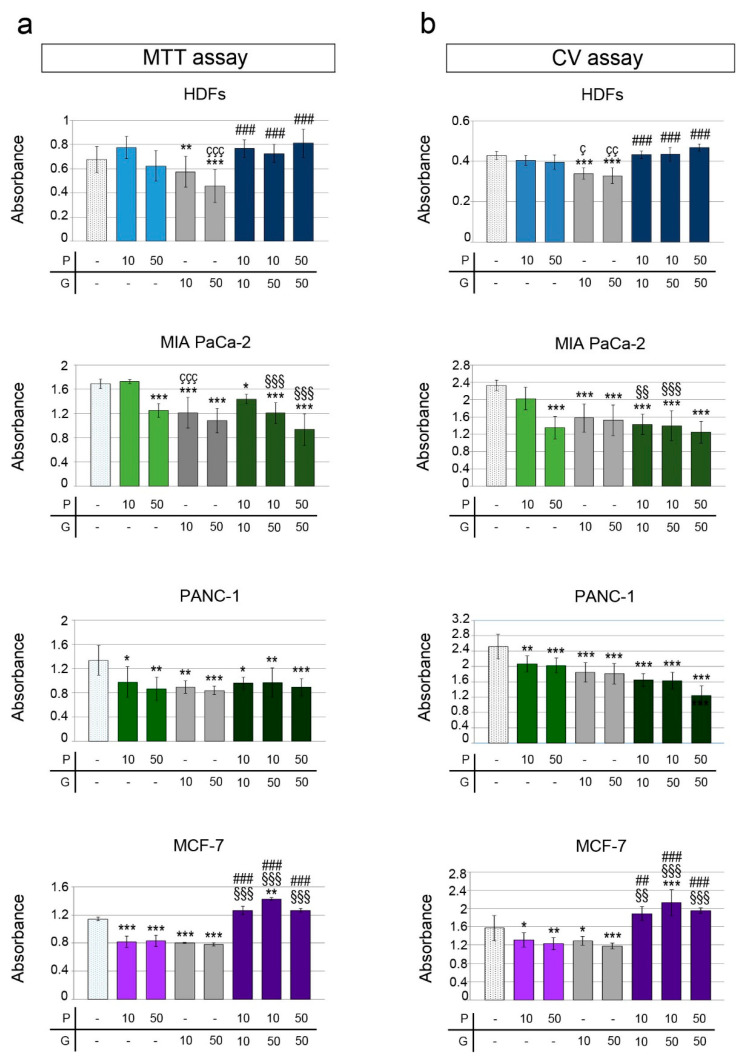
Impact of different PRO/gemcitabine (GEM) combinations on cell viability of HDFs, MIA PaCa-2, PANC-1 and MCF-7 cells. Cell viability of HDFs, MIA PaCa-2, PANC-1 and MCF-7 cells was assessed by MTT (**a**) or crystal violet (CV) assay (**b**). PRO and GEM doses (μM) used in treatments are indicated in *x*-axis. Graph columns represent mean of absorbance ± SD (**a**,**b**). The symbol * indicates statistical significance of any treatment versus control group; ç, statistical significance of GEM versus the corresponding concentration of PRO; §, #, statistical significance of PRO/GEM combinations versus the corresponding concentration of PRO or GEM, respectively. P = protopine; G = gemcitabine. *, ç *p* < 0.05; **, çç, §§, ## *p* < 0.01; ***, ççç, §§§, ### *p* < 0.001.

We then analyzed the effect of GEM and different combinations of PRO/GEM on cell viability in pancreatic MIA PaCa-2, PANC-1 and breast MCF-7 tumor cell lines, as well as in HDFs. Low GEM concentrations (0.05 μM, 0.25 μM or 1 μM) did not alter viability of MIA PaCa-2, MCF-7 cells and HDFs when compared to control (data not shown). Doses of GEM ≥10 μM exerted a cytotoxic effect on MIA PaCa-2, PANC-1, MCF-7 cells and HDFs (Figure 2a,b and Figure S2).

The 150 μM PRO dose exerted a hormetic effect on viability of HDFs when combined with different doses of GEM: in fact, when it was combined with a low dose (25 μM) of GEM, it significantly increased viability (134.1% ± 0.5%), while in combination with higher doses (50 or 150 μM) of GEM, decreased it (53.6% ± 0.8% or 56.7% ± 0.3%, respectively) (Figure S3).

The effect of the PRO/GEM combinations was different in MCF-7 breast cancer and pancreatic cancer cells (MIA PaCa-2 and PANC-1). In MCF-7 cells 10 μM PRO + 50 μM GEM or 50 μM PRO + 50 μM GEM treatments increased viability when compared to the single doses of PRO or GEM; in particular, 10 μM PRO + 50 μM GEM increased viability to 125.0 ± 2.0% (both using MTT and CV) (Figure 2a,b). The 150 μM PRO + 150 μM GEM combination significantly decreased cell viability (50.0 ± 8.6%), when compared to control, 150 μM PRO or 150 μM GEM (Figure S3), suggesting a hormetic effect of PRO/GEM treatments in these cancer cells.

In contrast to MCF-7, 10 μM PRO + 10 μM GEM or 10 μM PRO + 50 μM GEM treatment induced a significant viability decrease in MIA PaCa-2 cells when compared to control (85 ± 5% and 71 ± 10%, in MTT; 61 ± 10% and 60 ± 15% in CV, respectively). In addition, in both assays, 10 μM PRO + 50 μM GEM treatment induced a significant viability decrease when compared to 10 μM PRO.

Combined treatments did not induce any significant viability reduction in MIA PaCa-2 cells when compared to the corresponding doses of GEM in MTT and CV assay (Figure 2a,b). As demonstrated by MTT assay, the combination of 150 μM PRO with GEM (50 and 150 μM) decreased viability in MIA PaCa-2 (53 ± 7%, 28 ± 5%, respectively) when compared to control and GEM. Only the combination 150 μM PRO + 150 μM GEM reduced viability when compared to 150 μM PRO (Figure S3). The exposure to 150 μM PRO was more cytotoxic than the exposure to 150 μM GEM (Figure S3).

The different combinations of PRO/GEM had cytotoxic effects on the other pancreatic cell line, PANC-1. In particular, 10 μM PRO + 10 μM GEM, 10 μM PRO + 50 μM GEM or 50 μM PRO + 50 μM GEM decreased viability down to 85.0 ± 5%, 71 ± 10% and 55 ± 16% (MTT assay) and down to 61 ± 10%, 60 ± 15% and 54 ± 11%, (CV assay), compared to control (Figure 2a,b).

**Figure 3:**
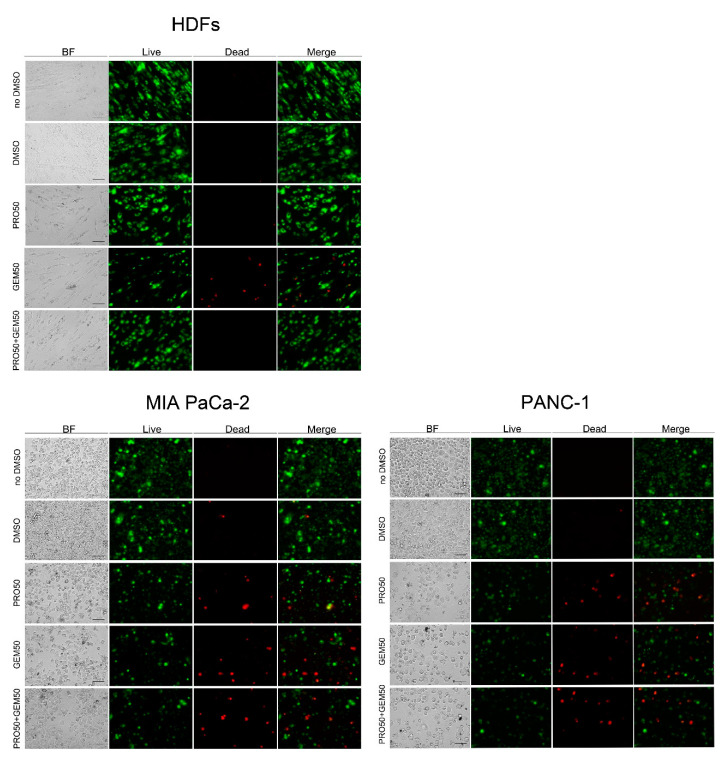
Fluorescence micrographs of LIVE/DEAD-stained HDFs, MIA PaCa-2, and PANC-1 cells. Cells were treated in the presence of 50 μM PRO, 50 μM GEM or 50 μM PRO + 50 μM GEM for 48 h or in the presence or absence of DMSO as vehicle (controls). Figures are representative images obtained with fluorescence microscopy, as reported in Materials and Methods. Green-labeled DiOC18(3)-positive cells correspond to live cells, red-labeled propidium iodide-positive cells correspond to dead cells. BF = brightfield. Scale bar = 100 μm, represented in BF images, is valid for all images.

The decrease in viability may be due to an impairment of mitochondrial function and/or a decrease in the number of cells. While MTT assay measures mitochondrial functionality, CV assay measures the incorporation of the stain into nucleic acids. After exposure to 10 μM PRO + 50 μM GEM, microscopic observations (Figure S4) showed a decrease in the density of adherent MIA PaCa-2 and PANC-1 cells, compared to controls. No noticeable changes were observed in MCF-7 cells and HDFs treated with the same combination or with 50 μM PRO + 50 μM GEM. In addition, by means of fluorescence microscopy we observed changes of viability after incorporation of the dye DiOC18(3) by living cells and of propidium iodide by dead cells (Figure 3).

In particular, after exposure to 50 μM PRO, 50 μM GEM or 50 μM PRO + 50 μM GEM, we observed a decrease of green-colored (live) as well as an increase of red-colored (dead) MIA PaCa-2 and PANC-1 cells, compared to controls (cells untreated or treated with DMSO only), while no visible changes in the number of green- and red-stained cells were observed for HDFs treated with 50 μM PRO or 50 μM PRO + 50 μM GEM when compared to controls. Diminution of live green-stained cells and increase of dead red-stained cells was detected following 50 μM GEM treatment when compared to controls. Notably, a *reduction* of the *dead cell* population after 50 μM PRO + 50 μM GEM treatment was observed (Figure 3). No apparent differences in red/green fluorescence between DMSO-treated and no DMSO-treated cells were observed.

### 2.3. Effects of PRO/GEM Combination on Cell Cycle Progression

**Figure 4:**
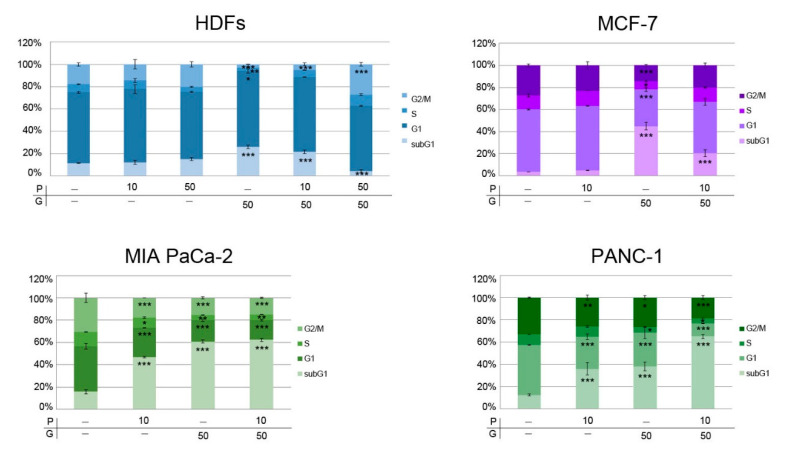
PRO/GEM combination altered cell cycle progression of MIA PaCa-2, PANC-1, MCF-7 cells and HDFs. The graphs show a quantification of cell cycle distribution, expressed as percent values of the means of cell number ± SD found in the cell cycle phases after different treatments. PRO and GEM doses (μM) used in treatments are indicated in x-axis. The symbol * indicates statistical significance versus control. P = protopine; G = gemcitabine. * *p* < 0.05, ** *p* < 0.01, *** *p* < 0.001.

Furthermore, we analyzed the effects of PRO, GEM and their combination on cell cycle progression in the different cell lines. In HDFs, PRO (10 μM or 50 μM) did not modify the cell cycle progression when compared to control. On the other hand, 50 μM GEM induced an increase of subG1 and G1, as well as a decrease of S and G2/M phases (Figure 4).

In HDFs, 10 μM PRO + 50 μM GEM treatment had effects similar to 50 μM GEM on G1 and G2/M phases; in addition, this treatment significantly (*p* < 0.05) increased the S phase compared to 50 μM GEM, restoring the control level. Remarkably, in these cells, the 10 μM PRO + 50 μM GEM or 50 μM PRO + 50 μM GEM treatments significantly reduced the mortality level (evaluated as subG1 phase) induced by 50 μM GEM (*p* < 0.05 and *p* < 0.001, respectively). In particular, the combination containing a higher concentration of PRO (50 μM PRO + 50 μM GEM) significantly decreased subG1 phase when compared to both control and 50 μM GEM. This result highlights the cytoprotective effect induced by this combined treatment in HDFs. Interestingly, the treatment with 50 μM PRO + 50 μM GEM increased G2/M phase when compared to control or 50 μM GEM (*p* < 0.001) (Figure 4). In MCF-7 cells, 10 μM PRO did not modify the cell cycle, whereas 50 μM GEM increased subG1 and decreased G1, S and G2/M phases. The combination 10 μM PRO + 50 μM GEM raised the number of cells in subG1 phase (563.0 + 13%) compared to control, but the number of cells decreased when compared to 50 μM GEM (*p* < 0.001). The combination 10 μM PRO + 50 μM GEM increased the percentage of MCF-7 cells in G1, S and G2/M compared to 50 μM GEM (*p* < 0.001, *p* < 0.05, *p* < 0.001, respectively) (Figure 4). These results suggest that the combination 10 μM PRO + 50 μM GEM increased cell death in this tumor cell line when compared to control.

The treatment with 10 μM PRO in MIA PaCa-2 and PANC-1 cells increased the percentage of cells in subG1 phase and decreased the number of cells in G1 and in G2/M phases. The cells in S phase were also reduced in number in MIA PaCa-2, but not in PANC-1 cells (Figure 4). In MIA PaCa-2 cells, the exposure to 50 μM GEM increased the percentage of cells in subG1 phase (387.1 ± 8.4%); however, the number of cells decreased in G1, S and G2/M (47.1 ± 1.8%, 36.1 ± 12.3 and 49.9 ± 3.2%, respectively). The combination 10 μM PRO + 50 μM GEM increased subG1 phase (396.5 ± 8.1%), and decreased G1, S and G2/M phases (43.4 ± 1.6%, 39.5 ± 2.5% and 48.7 ± 1.0%, respectively) when compared to control. In both cell lines this treatment did not alter cell cycle progression with respect to to 50 μM GEM (Figure 4). In PANC-1 cells, the incubation with 50 μM GEM increased the percentage of cells in subG1 phase (306.6 ± 30.8%), whereas it decreased the number of cells in G1, S and G2/M (67.6 ± 11.5%, 52.2 ± 1.9 and 80.3 ± 5.7%, respectively), compared to control. The combination 10 μM PRO + 50 μM GEM increased the subG1 phase (524.6 ± 13.2%), showing an additive effect when compared to 10 μM PRO (290.0 ± 45.5%); this combination decreased the number of cells in G1 and G2/M phases (26.0 ± 0.2% and 55.6 ± 4.9%, respectively), compared to control. In addition, the increase of number of cells in subG1 and their decrease in G1 phases, caused by 10 μM PRO + 50 μM GEM, appeared significantly higher than those induced by 10 μM PRO (*p* < 0.001) or 50 μM GEM (*p* < 0.001), while the decrease in G2/M phase had *p* < 0.05 and *p* < 0.01 compared to GEM and PRO, respectively (Figure 4). Taken together, these results show that the modification of cell cycle by PRO/GEM, when compared to GEM, was more evident in PANC-1 than in MIA PaCa-2 cells.

### 2.4. Effects of PRO and/or GEM on ROS Level

We evaluated the effect of PRO, GEM and PRO/GEM treatments on intracellular ROS levels on MIA PaCa-2, PANC-1, MCF-7 cell lines and HDFs by using two methods: (1) evaluation of fluorescence intensity of DCF (2,7-dichlorofuorescein diacetate) to measure hydroxyl, peroxyl and other ROS within the cell; (2) MitoSOX assay, a method widely used to detect mitochondrial ROS, especially superoxide [27,28,29].

#### 2.4.1. Intracellular ROS

**Figure 5:**
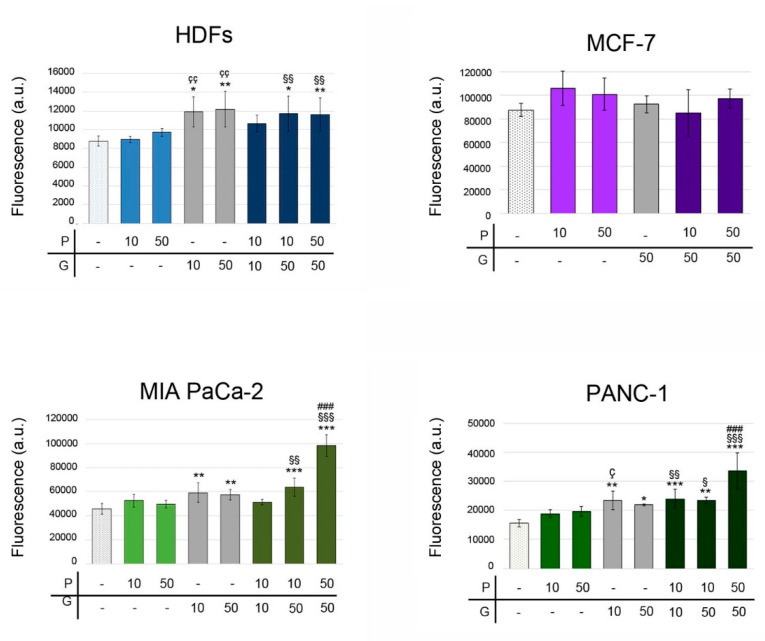
Impact of PRO/GEM treatments on intracellular ROS levels on MIA PaCa-2, PANC-1, MCF-7 cells and HDFs. The values of fluorescence intensity ± SD after PRO/GEM treatments are reported. The symbols denote statistical significance: *, any treatment versus control; ç, GEM versus the corresponding concentration of PRO; §, #, PRO/GEM combinations versus the corresponding concentration of PRO or GEM, respectively. P = protopine; G = gemcitabine. *, §, ç *p* < 0.05; **, §§, çç *p* < 0.01; ***, §§§, ###, *p* < 0.001.

Measurement of fluorescence indicated that GEM increased intracellular ROS production in HDFs, MIA PaCa-2 and PANC-1 cells, while PRO did not change ROS intracellular level in any of the analyzed cell lines (Figure 5).

In HDFs, the combined treatments 10 μM PRO + 50 μM GEM or 50 μM PRO + 50 μM GEM increased intracellular ROS levels (133.4 ± 21.6%, 132.4 ± 19.8%, respectively) when compared to control, but did not change when compared to GEM. In MCF-7 cells, the treatment with PRO (10 μM or 50 μM), 50 μM GEM, or the combination GEM/PRO did not alter the intracellular ROS levels compared to control (Figure 5). In MIA PaCA-2 and PANC-1, GEM increased intracellular ROS levels, 50 μM PRO + 50 μM GEM further increased them, while PRO did not have any effect. Also 10 μM PRO + 50 μM GEM and 50 μM PRO + 50 μM GEM treatments significantly increased ROS (139.4 ± 16.6%, 215.5 ± 19.8%, respectively) of MIA PaCa-2 cells, compared to control. The effect was similar in PANC-1 cells: 10 μM PRO + 10 μM GEM, 10 μM PRO + 50 μM GEM, or 50 μM PRO + 50 μM GEM increased ROS 153.8 ± 21.2%,150.6 ± 6.7%, 216 ± 39.7%, respectively (Figure 5). Interestingly, in both pancreatic cell lines, the 50 μM PRO + 50 μM GEM combination increased ROS levels also compared to 50 μM GEM (1.71- and 1.54-fold change, respectively) (Figure 5), showing a more than additive effect of this combination compared to GEM alone. Finally, intracellular ROS levels produced by the combined treatments appeared significantly increased when compared to the corresponding dose of PRO.

#### 2.4.2. Mitochondrial ROS

**Figure 6:**
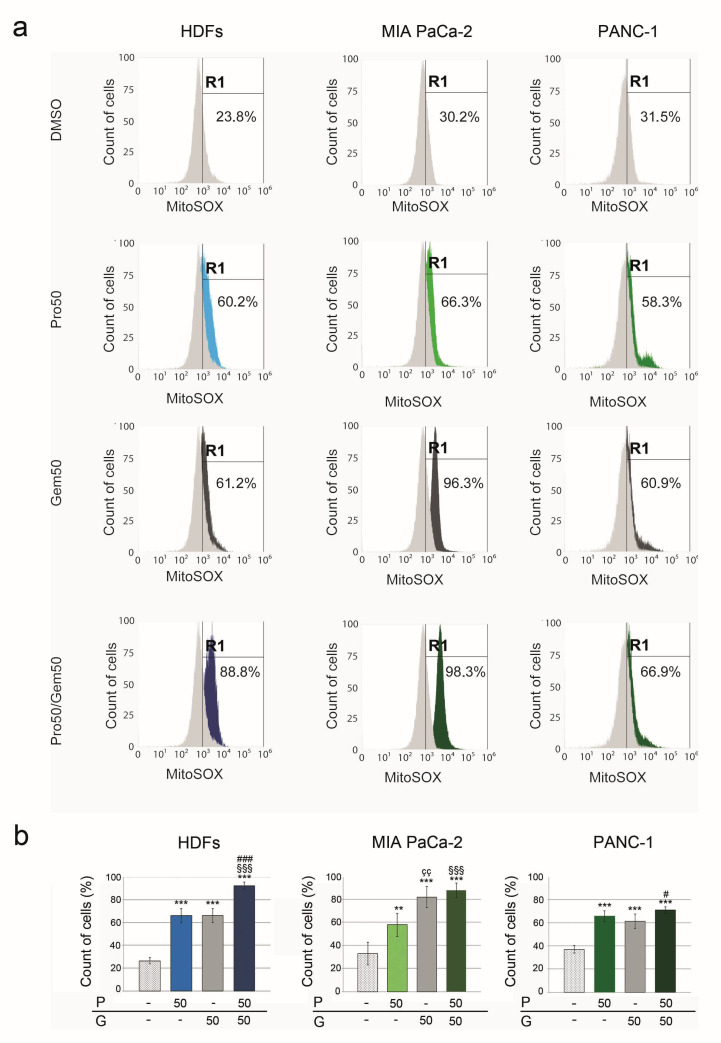
PRO, GEM and PRO/GEM increased mitochondrial ROS levels. (**a**) Flow cytometry analysis of MitoSOX signal in HDF, MIA Paca-2 and PANC-1 cells treated with DMSO, PRO50 μM, GEM50 μM or PRO50 μM + GEM50 μM. Measurements were performed using the BL3 (off 488 laser) 695/40 nm. *Y*-axis = percent of count of cells acquired (3 × 10^4^); *x*-axis = fluorescence intensity of the MitoSOX dye; R1 = gating strategy based on DMSO cell population used as a control. (**b**) Graph columns represent mean of MitoSOX signal ± SD, expressed as percent values. The symbol * denotes statistical significance versus control group; ç, statistical significance of GEM versus the corresponding concentration of PRO; §, #, statistical significance of PRO/GEM combination versus the corresponding concentration of PRO or GEM. P = protopine; G = gemcitabine. # *p* < 0.05; **, çç, *p* < 0.01; ***, §§§, ### *p* < 0.001.

MitoSOX-based assay demonstrated that PRO or GEM increased mitochondrial ROS in HDFs (250.4 ± 23.3% and 249.8 ± 23.4%, respectively) when compared to control (Figure 6a,b). PRO/GEM combination increased them (up to 350.7 ± 12.2%), showing an additive effect of the combination when compared to PRO or GEM alone. In MIA PACa-2 cells, mitochondrial ROS level increased to 175.6 ± 30.2%, 249.3 ± 28.4%, 267.3 ± 19.3% following PRO, GEM or PRO/GEM treatments, respectively. The increase of mitochondrial ROS level after PRO/GEM treatment was comparable to that observed in intracellular ROS under the same conditions (267.3 ± 19.3% versus 215.5 ± 19.8%). In PANC-1, mitochondrial ROS level increased to a similar extent following treatment with 50 μM PRO or 50 μM GEM (178.2 ± 12.7% and 166.1 ± 16.5%, respectively) while PRO/GEM had a higher effect (192.4 ± 7.4%) when compared to 50 μM GEM. Overall, our results showed that the treatments with PRO, GEM or PRO/GEM combination induced mitochondrial ROS production in HDFs, MIA PaCa-2 and PANC-1 cells. Although we observed no increase of fluorescence intensity after PRO treatment, FACS analysis of MitoSOX showed an increase of mitochondrial ROS. We hypothesize that the different sensitivity and selectivity of the methods for ROS detection may depend on their different subcellular resolution. Indeed, being a hydrophilic molecule, DCF can only penetrate the outer fenestrated mitochondrial membrane, while MitoSOX is able to enter the mitochondrial matrix.

## 3. Discussion

In this paper, we studied the effects of the natural alkaloid PRO and its combination with the chemotherapeutic drug GEM on cell viability, cell cycle progression and ROS levels in different tumor cell lines and HDFs. We found that the effect exerted by PRO was cell line-specific. In particular, PRO displayed higher cytotoxicity on pancreatic MIA PaCa-2, PANC-1 and breast MCF-7 tumor cells than on glioblastoma U343 and U87 cells. Interestingly, HDFs were more resistant to PRO. We also showed that the impact of PRO on cell cycle progression depended on cell type, PRO having a clear effect in the two pancreatic tumor cell lines, with a significant increase of cell death, while MCF-7 cells and HDFs were not affected. The resistance of HDFs to treatments could, at least in part, be explained by the action of multidrug resistance-associated proteins, including P-glycoprotein (Pgp, also known as MDR1) and MRP1 (MDR-associated protein 1), located in the cell membrane. In fact, MRP1 and Pgp confer resistance to a variety of anticancer drugs by acting as membrane pumps. Pgp has been suggested to regulate the efflux of tacrolimus in PBMCs (peripheral blood mononuclear cells) [30]. Furthermore, fibroblasts derived from *mrp1*(−/−) and *mdr1a/1b*(−/−) knock-out mice show increased sensitivity to vincristine and etoposide, compared to control fibroblasts. Finally, verapamil, an inhibitor of both MRP1 and Pgp, sensitized wild-type fibroblasts to both vincristine and etoposide [31].

Hormesis is a phenomenon of biphasic dose response in which a compound usually exhibits stimulatory or beneficial effects at low doses and inhibitory or toxic effects at high doses [32,33,34,35]. Hormetic effect should be considered in cancer therapy in order to optimize treatment. Our studies showed that PRO exerted a hormetic effect on PANC-1 cells, showing cytoprotection at low dose and cytotoxicity at higher doses. It is known that several tumor cell lines displayed biphasic dose responses following treatments with a wide range of agents including phytocompounds [33,34,35]; some molecular mechanisms underlying hormesis have been investigated in cultured cells and very few in animal models [33,34,35]. Our results are in line with previous studies reporting antiproliferative activity of PRO in MCF-7, cervical cancer HeLa cells, human osteosarcoma U2OS cells, prostate PC-3 and breast MDA-MB-231 cancer cells, and low or no cytotoxicity in non-tumor cells, such as HUVECs and lipopolysaccharide-activated murine macrophages (Raw 264.7 cells) [12,21,23,36]. In particular, PRO affected microtubule structures in HeLa and U2OS cells, promoted G2/M arrest of cell cycle in HeLa cells and induced apoptosis in HCT116 colon cancer cells by activating the p53 pathway [23,37]. In addition, extracts of *Chelidonium majus* L. containing PRO exhibited a cell line-dependent effect, showing cytotoxicity on PANC-1, HT-29 and MDA-MB-231 tumor cells, but only low or no cytotoxicity in primary endometrium cancer cells and murine normal 3T3 fibroblasts [13]. Finally, PRO had a protective effect on lipopolysaccharide-induced acute kidney injury in mice by reducing white blood cell counts and ROS [38].

PRO/GEM combinations were cytotoxic or cytoprotective depending on concentrations and cell type. Some combinations showed a cytoprotective effect in HDFs but did not modify the GEM-induced cytotoxicity on MIA PaCa-2 and PANC-1 cells. The cytotoxic effect in pancreatic tumor cell lines was confirmed by cell cycle analysis. In MIA PaCa-2 cells, the number of dead cells (measured as subG1 phase) was similar in GEM and PRO/GEM treatments, whereas in PANC-1 the effect of PRO and GEM was additive, since PRO/GEM treatment further increased cell death induced by GEM. It is known that GEM increases apoptotic cell death and activates caspase 3 in both MIA PaCa-2 and PANC-1 cell lines [39,40,41]. Moreover, PRO increases the mitochondrial apoptotic pathway in human hormone-refractory prostate cancer cells [21]. Whether the subG1 population corresponds to apoptotic or other types of cell death in MIA PaCa-2 and PANC-1 cells needs further investigation.

Overproduction of ROS causes oxidative stress that is harmful to cell structures and may activate cell death processes [42]. Induction of ROS by GEM is considered one of the mechanisms for its antitumor activity [6]. Notably, GEM treatments increased the level of intracellular ROS, including mitochondrial ROS, in pancreatic MIA PaCa-2 and PANC-1 tumor cells. This is consistent with previous reports in PDAC cells [6,43]. We have found that 50 μM PRO + 50 μM GEM combination induces a higher intracellular ROS increase than GEM alone in MIA PaCa-2 and PANC-1 cells (up to 2-fold), suggesting the involvement of ROS in the mechanism of PRO/GEM cytotoxicity. The increase of mitochondrial ROS level induced by 50 μMPRO + 50 μM GEM treatment supports this possibility. We detected an increase of mitochondrial ROS, but not of intracellular ROS, in HDFs, MIA PaCa-2 and PANC-1 cells after 50 μM PRO treatment. This could depend on the lower sensitivity of the DCF fluorescence detection method. We hypothesize that intracellular ROS scavenging mechanisms may attenuate the mitochondrial superoxide increase due to PRO treatment [44]. This could explain why no ROS alteration was detected outside the mitochondrial matrix by DCF, a molecule that is unable to cross the inner mitochondrial membrane. It is known that alkaloids, such as PRO, act as antioxidant agents [45,46]. For example, pretreatment with PRO significantly decreased lactic dehydrogenase activity and increased superoxide dismutase activity in serum of ischaemic rats, suggesting that PRO neuroprotective effects can be related to its antioxidant property [47]. In PC12 cells, PRO has a cytoprotective action by promoting an antioxidative response, with reduction of H_2_O_2_-induced oxidative stress and cell death [36]. Similarly, antioxidative mechanisms could scavenge the ROS mitochondrial increase induced by PRO and protect HDFs from cell death. The excess of mitochondrial ROS elicited by PRO in HDFs could be associated with senescence induction. In fact, mitochondrial ROS alteration can occur as a cell response towards age-dependent damage [48]. The ROS scavenger system is also deteriorated in aged cells, shifting aged cells towards a pro-oxidant status [48]. Although it is not known whether PRO causes senescence in HDFs, experimental evidence shows that other isoquinoline alkaloids, such as berberine, can produce senescence-like growth arrest in these cells [17].

The increase of mitochondrial ROS observed in MIA PaCa-2 and PANC-1 cells by 50 μM PRO treatment suggests that PRO induces cytotoxicity via a ROS-mediated mechanism in these cell lines. On the whole, these results indicate that PRO exhibits pro-oxidant or antioxidant properties, depending on dose and cell type [26,49]. Recent evidence demonstrates that prolonged chemotherapy reduces the overall cellular ROS in cancer, and this reduction may contribute to the mechanism of drug resistance [50,51], including that of PDAC to GEM [52]. For these reasons, to avoid chemoresistance, the use of compounds able to overcome the antioxidant mechanisms and to promote high levels of ROS represents one promising therapeutic strategy to induce cancer cell death and inhibit cancer progression [53,54,55]. Our results highlight the oxidative properties of PRO in MIA PaCa-2 and PANC-1 and imply that PRO may be a useful adjuvant of GEM in anticancer therapy.

The vitality assays and cell cycle analysis show that in MCF-7 cells, most of the PRO/GEM combinations had a cytoprotective action and only 150 μM PRO + 150 μM GEM was more cytotoxic than GEM or PRO alone. Although we did not detect an intracellular ROS increase in MCF-7 in our experimental conditions, we cannot exclude an increase of mitochondrial ROS, undetected by the DCF method. In fact, it has been demonstrated that a higher GEM concentration (1 mg/mL) disrupts mitochondrial membrane potential and increases ROS levels in MCF cells [56].

As in MCF-7 cells, in HDFs GEM action was significantly cytotoxic, while 50 μM PRO + 50 μM GEM was cytoprotective, by increasing the G2/M phase of cell cycle and by reducing mortality. PRO alone did not alter the HDFs viability, but a cytotoxic effect occurred with most combinations containing a higher dose of PRO (150 μM).

Notably, HDFs exhibited a biphasic dose response to PRO (150 μM)/GEM treatments, indicating that, similarly to other natural compounds and also to some chemotherapeutics [32,33,34,35], PRO in combination with GEM may induce a hormetic effect. In addition, PRO/GEM treatments caused a hormetic response in MCF-7 cancer cells, suggesting a cautious use of natural compounds as anti-cancer agents.

Notably, PRO/GEM treatments caused both intracellular and mitochondrial ROS increase in HDFs (up to 350.7 ± 12.2%) without causing viability decline. Because normal cells produce less ROS than cancer cells and have efficient antioxidant systems [50], it is possible that an antioxidant response is activated in HDFs following the ROS induction due to PRO/GEM combinations. Further experiments will be necessary to confirm the oxidative stress resistance of these cells.

Notably, MIA PaCa-2, PANC-1, MCF-7 cells and HDFs differ in the state of *p53* gene: MCF-7 and HDFs cells have a wild-type *p53*, while MIA PaCa-2 cells have a *p53* homozygous missense mutation in exon 7. Finally, PANC-1 cells have two homozygous missense mutations in exons 4 and 8 of this gene [57]. The alteration of *p53* in tumor cells often leads to the acquisition of properties promoting tumor progression, such as drug resistance, hyperproliferative capacity and invasiveness, while the inhibition of the mutated p53 protein attenuates these features [58,59]. However, this is still a debated question. In fact, it has been shown that tumor cells with wild-type *p53* gene show resistance to pharmacological therapy [60,61]. On the other hand, some reports show that apoptotic response to 5-fluorouracil was significantly reduced in HCT116 cells expressing mutant *p53*, compared to cells expressing wild-type *p53* [62]. MCF-7 cells (with wild-type *p53*) showed no increase in apoptosis after 72 h of GEM treatment [1]. Conversely, MCF-7/Adr cells (with a mutated *p53* gene) have a significant increase in apoptosis after 48 h of exposure. We speculate that the different status of *p53* could influence the response of MIA PaCa-2, PANC-1, MCF-7 cells and HDFs to PRO/GEM treatment.

Taken together, our results suggest that PRO (10 μM, 50 μM) can be used in combination with GEM to protect fibroblasts from the effect of chemotherapy, without altering GEM cytotoxicity versus pancreatic tumor cells. Recent data indicate new roles of fibroblasts in tumorigenesis. For example, pancreatic cancer is characterized by the presence of a stromal component, including fibroblasts and extracellular matrix, whose role is still controversial [63,64,65,66,67]. Some studies report a tumorigenic role for this component [68], while others have shown a protective effect of desmoplastic stroma in preventing tumor proliferation and invasion in PDAC [69,70]. Interestingly, ROS have emerged as key regulators of the reciprocal signaling between PDAC and the surrounding stromal microenvironment [71]. Therefore, further increasing the elevated ROS levels to induce cytotoxicity has produced antitumorigenic effects. In addition, manipulation of stromal cells for drug delivery represents an approach to circumvent pancreatic tumor cell resistance to chemotherapy. Indeed, human stromal dermal fibroblasts incorporated paclitaxel and then released it with unaffected pharmacological activity, thus inhibiting human IgR39 melanoma growth in vitro [72]. Since we found that PRO in combination with GEM exerted a beneficial effect on fibroblasts, it will be crucial to analyze whether this combination has a protumorigenic or antitumorigenic effect in PDAC cells co-cultured with fibroblasts; further experiments will be necessary to dissect this point as well as the molecular mechanisms involved in the effects induced by PRO/GEM. To assess the hypotheses emerging from our studies, it will be necessary to test the effects of PRO/GEM combinations in in vivo pancreatic cancer models.

## 4. Materials and Methods

### 4.1. Cell Culture

In this study, we used tumor cell lines (MIA PaCa-2, PANC-1, U343, U87, MCF-7) and non-tumor cells (HDFs) as a control. The human cancer cell lines that were used in this study were pancreatic carcinoma cells (MIA PaCa-2 and PANC-1; American Type Culture Collection, ATCC), human glioblastoma cells (U343 and U87 MG; CLS Cell Lines Service), breast cancer cells (MCF-7; ATCC) and normal primary dermal fibroblasts HDFs (ATCC, Manassas, VA, USA). Cells (MIA PaCa-2, PANC-1, U343, MCF-7 and HDFs) were grown in DMEM (Dulbecco’s modified eagle’s medium high glucose, Sigma-Aldrich, Milan, Italy) or in RPMI-1640 (U87 cells) medium (Sigma-Aldrich), supplemented with 10% FBS (fetal bovine serum; Sigma-Aldrich), 100 U/mL penicillin and 100 mg/mL streptomycin (Gibco, Thermo Fisher Scientific, Waltham, MA, USA), 1% MEM non-essential amino acid solution (Sigma-Aldrich), 1% l-Glutamine 200 mM (Sigma-Aldrich) (complete media). All cell lines were grown and maintained at 37 °C in humidified air with 5% CO_2_.

### 4.2. Cell Treatments

Cell lines were treated for 48 h with different concentrations (0.003–300 µM) of PRO hydrochloride (Sigma-Aldrich) and/or (0.05–150 μM) of GEM hydrochloride (Sigma-Aldrich), or with different combinations of PRO and GEM as indicated in the experiments. PRO and GEM dilutions were obtained from a 10^−2^ M stock solution prepared in DMSO (Sigma-Aldrich). For the combined treatments, PRO and GEM were simultaneously added to complete medium. Control cells were treated with corresponding volumes of DMSO. The treatments were renewed every 24 h.

### 4.3. Cell Viability Assays

#### 4.3.1. MTT Assay

All the cell lines were seeded onto 96-well flat bottom microplates treated as described above when they reached 60% confluence and analyzed by Cell Proliferation Kit I MTT assay (Roche, Monza, Italy). The MTT assay was performed according to the manufacturer’s instructions and absorbance at 570 nm was measured using an Ultra Microplate reader (Bio-Tek Instruments Inc., Winooski, VT, USA).

#### 4.3.2. Crystal Violet (CV) Assay

For the CV assay, cells were seeded and treated as above and stained with 1% crystal violet (Sigma-Aldrich) for 30 min at 37 °C. Microplates were washed by submersion in flowing tap water for 15 min, allowed to air dry overnight and incubated with 10% acetic acid for 15 min at room temperature with gentle shaking to dissolve the dye. Then, the absorbance at 595 nm was measured using an Ultra Microplate reader (Bio-Tek Instruments Inc.).

#### 4.3.3. LIVE/DEAD Cell Cytotoxicity Assay

Viability was also evaluated with the “LIVE/DEAD Cell” cytotoxicity kit according to the manufacturer’s protocol (Molecular Probes, Life Technologies, Carlsbad, CA, USA). Cells were plated in 8-well chambers on coverslip II (Sarstedt, Nümbrecht, Germany) at 25,000 cell/cm^2^ (MIA-PaCa-2 and PANC-1 cells) or 9400 cell/cm^2^ (HDF) and treated with 50 μM PRO, 50 μM GEM or 50 μM PRO + 50 μM GEM in complete DMEM without phenol red for 48 h. Untreated cells (no DMSO) and DMSO-treated cells were used as controls. New medium containing the appropriate additives and 12 μM DiOC18(3) (3,3-dioctadecyloxacarbocyanine perchlorate) was added after 24 h. On the next day, cells were washed with phosphate buffered saline (PBS) and counterstained with 7.5 μM propidium iodide in DMEM without phenol red and observed with a fluorescence microscope EVOSFLoid Cell imaging station (Thermo Fisher Scientific, Waltham, MA, USA) according to manufacturer’s protocol.

### 4.4. Cell Cycle Analysis

Cells were seeded onto 10 mm plates (5 × 10^4^ cells/cm^2^), treated as above and analyzed for cell cycle progression. Cell cycle distributions were determined using propidium iodide (Sigma-Aldrich) staining and flow cytometry, as reported previously [17]. After treatments, MIA PaCa-2, PANC-1, MCF-7 cells and HDFs were washed with PBS (Sigma-Aldrich), trypsinized, fixed in 95% cold ethanol diluted 3:1 in PBS and stored at 4 °C overnight. Cell pellets were washed twice with PBS, resuspended in staining solution (50 μg/mL propidium iodide, 0.1% sodium citrate, 0.5 mg/mL RNase A and 0.1% Nonidet NP40) and incubated overnight at 4 °C. Cell cycle distributions were determined using flow cytometry (BD FACSJazz™ Cell Sorter, BD Bioscience, San Jose, CA, USA) and data were analyzed using BD FACS Software (BD Bioscience).

### 4.5. ROS Detection

Cellular and mitochondrial ROS generation by MIA PaCa-2, PANC-1, MCF-7 cells, and HDFs was detected by using DCF (Sigma-Aldrich) and MitoSOX (Life technologies, Monza, Italy) probes, respectively.

#### 4.5.1. ROS Detection with the Fluorescent Probe DCF

Cells were seeded at the density of 5 × 10^4^ cells/well in 96 well-black plates with flat bottom and, following treatments, were rinsed with PBS and incubated with 15 µM DCF for 30 min at 37 °C. ROS production was measured by fluorescence intensity using a Victor X3 Multilabel Plate Reader (excitation 485 nm, emission 535 nm).

#### 4.5.2. Flow Cytometry Analysis of MitoSOX Signal

PANC-1, MIA PaCa-2 (200,000 in 60 mm diameter plates) and HDF cells (150,000 in 100 mm diameter plates) were incubated for 48 h with 50 µM PRO, 50 µM GEM, 50 µM PRO + 50 µM GEM or vehicle. Then, cells were stained with 1 µM MitoSOX Red (Thermo Fisher Scientific) for 40 min at 37 °C following manufacturer’s instructions. After staining, cells were washed with PBS, trypsinized and analyzed by flow cytometry using the ATTUNE NTX Flow Cytometer (Thermo Fisher Scientific). The number of events acquired for all testing by flow cytometry was fixed at 3 × 10^4^ cells.

### 4.6. Statistical Analysis

Data are expressed as mean ± SD of at least three independent experiments with at least three replicates. Normality and homoscedasticity were tested. Statistical significance was determined by one-way ANOVA followed by a Tukey’s multiple comparison test, with Statgraphics (version XVI) and GraphPad Prism 6 software. *p* < 0.05 was considered to be statistically significant.

## 5. Conclusions

In summary, in this paper we show that: (1) the response to PRO treatments was cell line-specific; (2) the different treatments with PRO induced greater cytotoxic effects in MIA PaCa-2, PANC-1, U343, U87 and MCF-7 tumor cells, compared to non-tumor HDFs; (3) PRO induced a hormetic response on PANC-1 viability; (4) the PRO/GEM combination acted in dose-dependent and cell-specific manner, determining a cytotoxic effect on MIA PaCa-2 and PANC-1 cells. High doses of PRO in combination with GEM were cytotoxic in HDFs and MCF-7 cells, whereas lower doses attenuated the cytotoxicity of GEM, indicating that this alkaloid may induce hormetic effects.

## Data Availability

Te datasets generated during and/or analysed during the current study are available from the corresponding author on reasonable request.

## Supplementary Materials

The following are available online at https://www.mdpi.com/1424-8247/14/2/90/s1, Figure S1: PRO differentially affects the viability of MIA PaCa-2, PANC-1, U343, U87, MCF-7 and HDFs, Figure S2: Impact of GEM treatments on MIA PaCa-2, PANC-1, MCF-7 cells and HDFs, Figure S3: Impact of 150 μM PRO combined with different doses of GEM on cell viability of HDFs, MIA PaCa-2 and MCF-7 cells (MTT assay), Figure S4: MIA PaCa-2, PANC-1, MCF-7 cells and HDFs after PRO/GEM treatments.
