# Supplementary material for: Protopine/Gemcitabine Combination Induces Cytotoxic or Cytoprotective Effects in Cell Type-Specific and Dose-Dependent Manner on Human Cancer and Normal Cells
Source: Pharmaceuticals (Basel). 2021 Jan 26;14(2):90. doi: 10.3390/ph14020090 (PMC7912662; doi:10.3390/ph14020090)
Supplement: Supplementary file 1 [file pharmaceuticals-14-00090-s001.pdf]

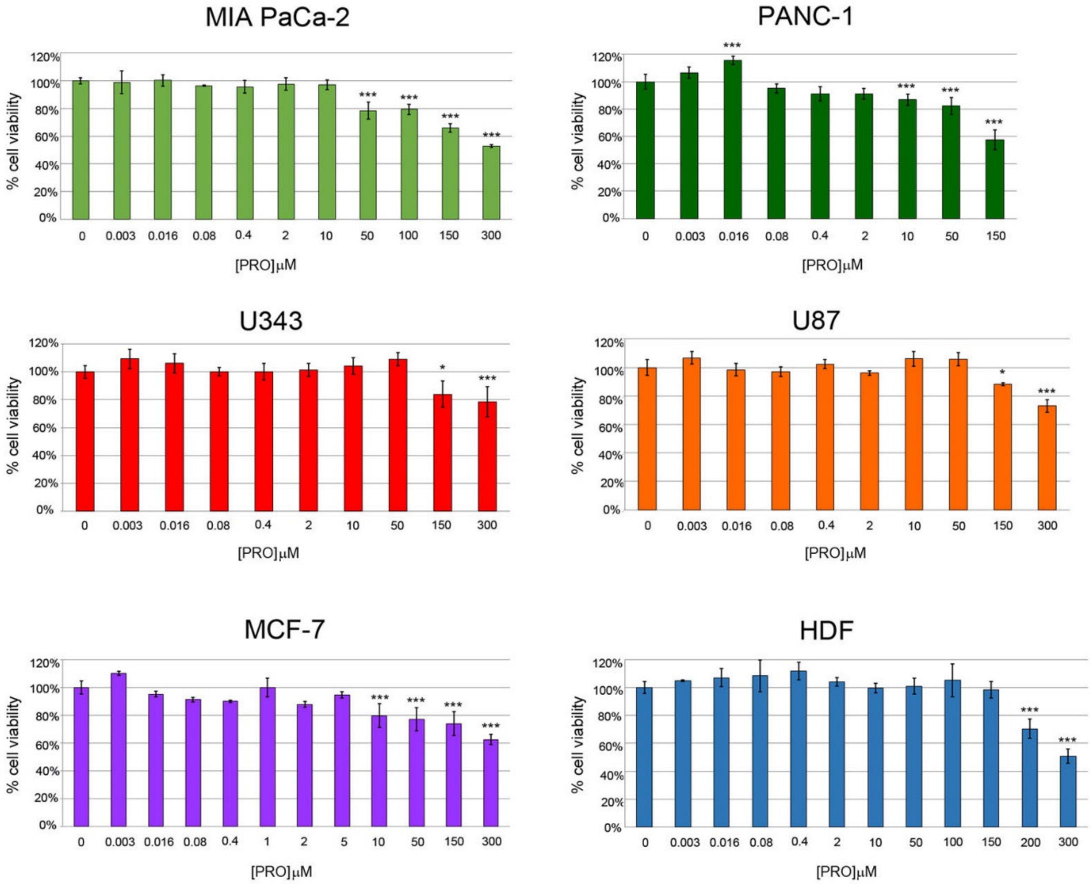

Figure S1. PRO differentially affects the viability of MIA PaCa-2, PANC-1, U343, U87, MCF-7 and HDFs. Cell viability was evaluated after treatments with different PRO doses, by using MTT. Graph columns represent mean of viable cells ± SD normalized versus control group. The PRO doses (μg) used in treatments are indicated in x-axis. The symbols * indicate statistical significance versus DMSO. * $p < 0.05$; ** $p < 0.01$; *** $p < 0.001$.

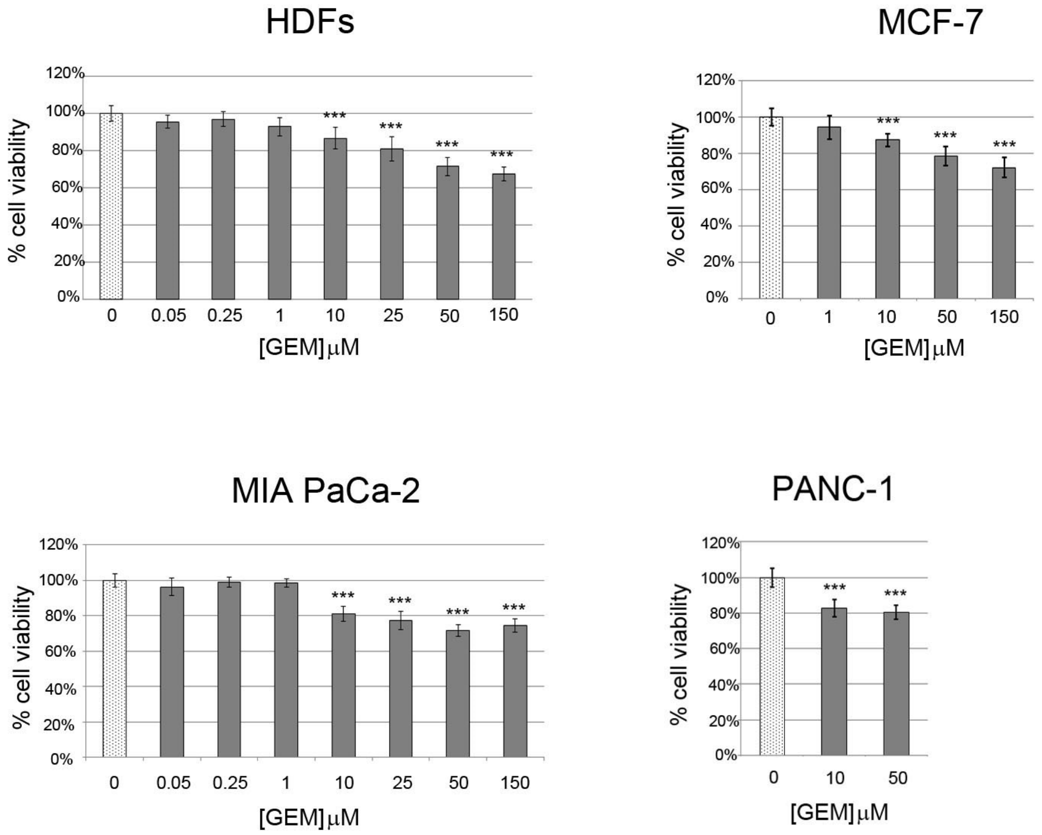

Figure S2. Impact of GEM treatments on MIA PaCa-2, PANC-1, MCF-7 cells and HDFs. Changes of cell viability (MTT assay) have been detected after treatments with different GEM doses (μM) indicated in x-axis. Graph columns represent mean of viable cells ± SD normalized versus control. * $p < 0.05$; ** $p < 0.01$; *** $p < 0.001$.

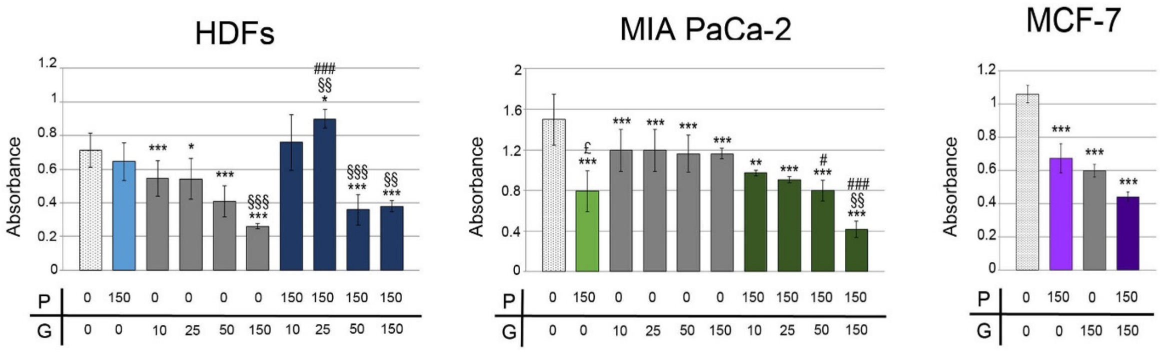

Figure S3. Impact of 150µM PRO combined with different doses of GEM on cell viability of HDFs, MIA PaCa-2 and MCF-7 cells (MTT assay). PRO and GEM doses (µM) used in the treatments are indicated in x-axis. Graph columns represent mean ± SD of absorbance values. The symbol * indicates statistical significance versus control. The symbols §, # indicate statistical significance of PRO/GEM combinations versus the corresponding concentration of PRO or GEM, respectively. P= protopine; G= gemcitabine. *, §, # $p < 0.05$; **, §§, ## $p < 0.01$; ***, §§§, ### $p < 0.001$.

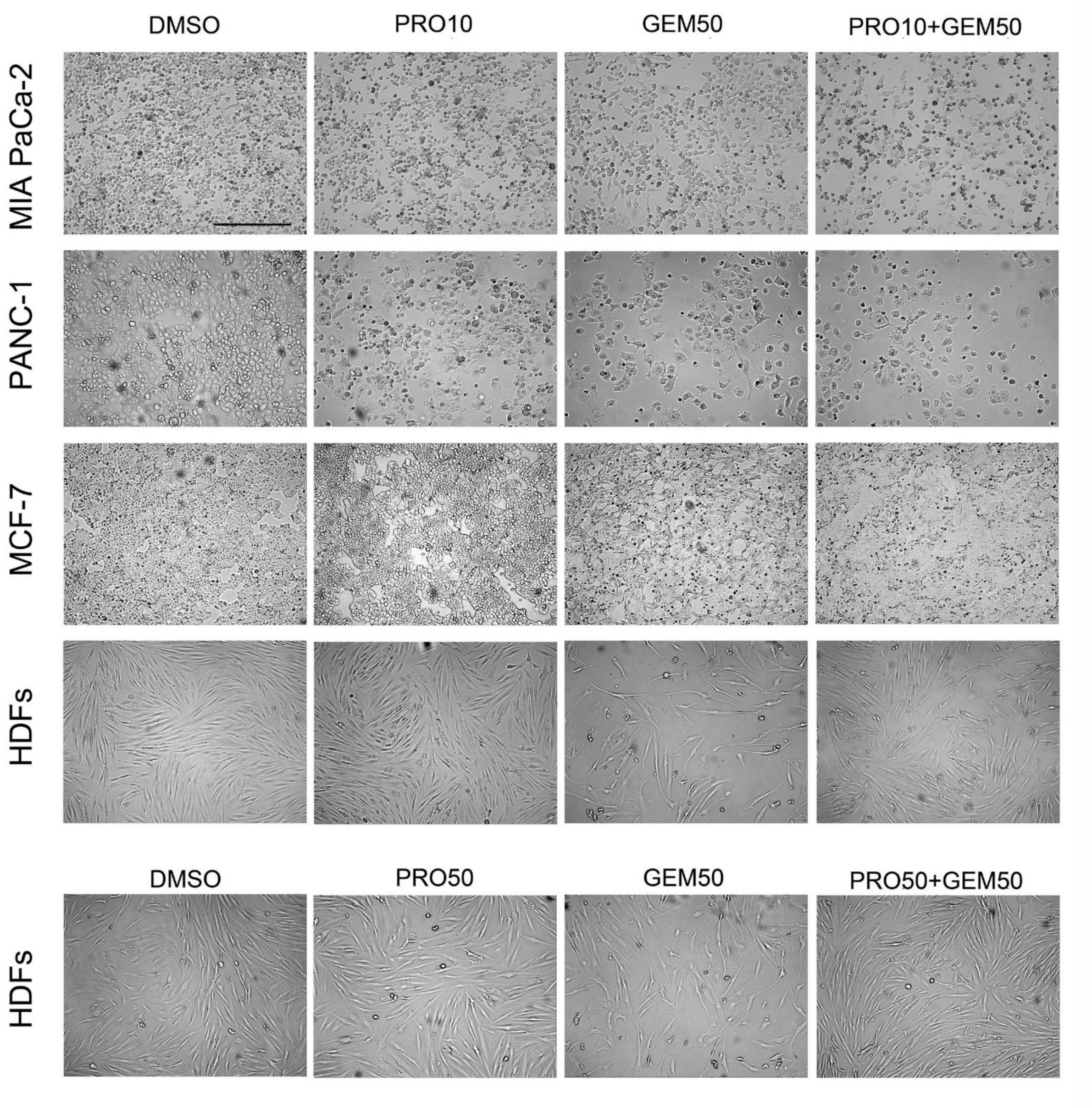

Figure S4. MIA PaCa-2, PANC-1, MCF-7 cells and HDFs after PRO/GEM treatments. The images show the cellular density of MIA PaCa-2, PANC-1, MCF-7 cells and HDFs. After exposure to 10μM PRO+50μM GEM, a decrease in the density of MIA PaCa-2 and PANC-1 cells was evident, compared to control. No apparent change of cell density has been observed in HDFs. Scale bar = 500 μm, represented in MIA PaCa-2 treated with DMSO, is valid for all images.
